# Analyzing lipid profiles and dyslipidemia prevalence in hypertensive patients: a cross-sectional study from primary community health institutions

**DOI:** 10.3389/fmed.2024.1425414

**Published:** 2024-12-17

**Authors:** Wenxin Wang, Xinmin Li, Deliang Lv, Xiaobing Wu, Fengzhu Xie, Wei Xie, Jinxiao Wang, Zhiguang Zhao

**Affiliations:** ^1^School of Public Health/Institute of Local Government Development, Shantou University, Shan-Tou, China; ^2^School of Public Health, Shantou University, Shan-Tou, China; ^3^Shenzhen Center for Chronic Disease Control, Shenzhen, China

**Keywords:** dyslipidemia, epidemiology, lipid level, prevalence, dyslipidemia component, associated factors

## Abstract

**Background:**

A significant proportion of hypertensive patients also suffer from comorbid dyslipidemia, which critically influences their treatment outcomes and overall prognosis. Given its implications, the lipid profiles of hypertensive individuals warrant increased attention for more effective clinical management.

**Methods:**

We analyzed data from 92,443 hypertensive patients registered at primary community health institutions in 2021. Employing a cross-sectional study design, we assessed the distribution of lipid levels and the prevalence of various dyslipidemia subtypes. Stepwise forward logistic regression was used to identify factors associated with dyslipidemia, adjusting for gender, age, body size, and other relevant characteristics.

**Results:**

According to the 2023 Chinese Guidelines for the Management of Lipids, the overall prevalence of dyslipidemia was 37.5%. Subtype analysis revealed prevalence of high total cholesterol (TC) at 11.2%, high triglycerides (TG) at 16.0%, low high-density lipoprotein cholesterol (HDL-C) at 16.0%, and high low-density lipoprotein cholesterol (LDL-C) at 10.2%. TG abnormalities were more common among males (16.8%), whereas TC abnormalities predominated in females (14.4%). Notably, hypertensive patients with diabetes had higher levels of TG compared to non-diabetics (*p* = 0.009). Those with stroke and liver disease comorbidities exhibited lower TG levels than their counterparts (*p* = 0.018 and *p* < 0.001, respectively). Additionally, HDL-C levels were significantly lower in hypertensives with diabetes, coronary artery disease, and central obesity (*p* < 0.001, *p* = 0.026, p < 0.001, respectively). Regression analysis indicated that dyslipidemia prevalence correlates significantly with gender, age, diabetes, coronary heart disease, stroke, family history of hypertension, body mass index (BMI), central obesity, frequency of physical activity, smoking status, regular alcohol consumption, and abdominal ultrasound findings.

**Conclusion:**

Our study underscores the necessity of rigorous lipid monitoring and analysis of dyslipidemia-influencing factors for the development of effective health management strategies within the community. There is a critical need to examine lipid profiles comprehensively and implement targeted therapeutic interventions aimed at managing hyperlipidemia, a modifiable risk factor for cardiovascular disease.

## 1 Introduction

Dyslipidemia has been identified as a major risk factor for cardiovascular disease (CVD) (1, 2). Over the last three decades, the prevalence of dyslipidemia and lipid levels has continued to rise in China. Elevated serum cholesterol levels are estimated to lead to an increase of approximately 9.2 million cardiovascular events in the Chinese population between 2010 and 2030 (3). Furthermore, elevated low-density lipoprotein cholesterol (LDL-C) was the third most significant risk factor for cardiovascular mortality in China, according to the 2017 Global Burden of Disease Study (4). This study also attributes approximately one-third of cardiovascular deaths in China to elevated LDL-C. Dyslipidemia, particularly characterized by elevated LDL-C or total cholesterol (TC), is a critical risk factor for atherosclerotic cardiovascular disease (ASCVD). Evidence shows that lowering LDL-C levels significantly reduces the morbidity and mortality associated with ASCVD (5, 6).

In China, there are approximately 245 million people with hypertension. It is projected that by 2025, the global number of hypertensive patients will increase to 1.56 billion. Even among patients with well-controlled blood pressure, the risk of cardiovascular disease significantly escalates with elevated LDL levels. The coexistence of dyslipidemia and hypertension geometrically increases the risk of cardiovascular disease. Both conditions are important, controllable risk factors for CVD. As such, managing lipid health alongside blood pressure is crucial for the prevention and treatment of cardiovascular and cerebrovascular diseases (7, 8).

Lipid management has become a major public health concern in China. Given the high prevalence of dyslipidemia among hypertensives, it is imperative to focus on this demographic. Understanding current dyslipidemia trends among hypertensive patients in community primary health institutions, and implementing targeted preventive measures and interventions, are essential for improving treatment efficacy in this group (9, 10). This study aims to investigate the prevalence of dyslipidemia and its influencing factors among hypertensive patients in Shenzhen, to provide a theoretical basis for improving hypertension health management and services, and to explore a region-specific pattern of dyslipidemia management in primary public health setting.

## 2 Materials and methods

### 2.1 Study design and procedure

This study employed a cross-sectional survey design targeting hypertensive patients enrolled in the Basic Public Health Hypertension Management and Service program in Shenzhen, which is part of the national Basic Public Health Service programs. These programs are dedicated to providing healthcare services to hypertensive patients. The final analysis included data from a total of 92,443 respondents.

Inclusion Criteria: Eligible participants were those (1) diagnosed with hypertension and registered under the Basic Public Health Service, and (2) who had completed health examinations with comprehensive blood lipid tests including total cholesterol (TC), high-density lipoprotein cholesterol (HDL-C), triglycerides (TG), and low-density lipoprotein cholesterol (LDL-C).

Exclusion Criteria: The study excluded individuals (1) with incomplete resident health records or examination results, and (2) missing values for any lipid measurements.

### 2.2 Data collection

Data collection involved retrieving medical health records and annual health examination information from primary community health centers. All hypertensives under the Basic Public Health Hypertension Management and Service program participated in follow-up clinics for hypertension and underwent an annual health physical examination at these centers. Disease comorbidities were collected through self-reported previous disease history and family history of disease during treatment. For instance, diabetes was identified in individuals who self-reported a history of a diabetes diagnosis or who had a fasting blood glucose test result greater than or equal to 7.0 mmol/L. A family history of diabetes was noted for those reporting diabetes in parents or siblings. Additionally, basic socio-demographic characteristics (such as gender, age, marital status, type of residence, body mass index, waist circumference), behavioral lifestyle factors (physical exercise, smoking status, alcohol consumption status), medical history (diabetes, coronary heart disease, stroke, cancer, hepatic disease), physiological and biochemical indicators (SBP, DBP, TC, TG, HDL-C, LDL-C), and other relevant information were collected.

### 2.3 Definition of terms

According to the Chinese Guidelines for the Management of Lipids (2023), a dyslipidemia is defined as the presence of any of the following conditions: (1) TC ≥ 6.2 mmol/L, (2) TG ≥ 2.3 mmol/L, (3) HDL-C < 1.0 mmol/L, (4) LDL-C ≥ 4.1 mmol/L. Body mass index is calculated based on relevant weight and height. According to the Chinese Guidelines for the Prevention and Control of Overweight and Obesity in Adults: Underweight is BMI < 18.5 kg/m^2^, normal weight is 18.5 kg/m^2^ ≤ BMI < 24.0 kg/m^2^, overweight is 24.0 kg/m^2^ ≤ BMI < 28.0 kg/m^2^, obese is BMI ≥ 28.0 kg/m^2^. Central obesity is waist circumference > 90 cm for men and > 85 cm for women. Physical activity frequency was categorized as daily, regularly and less frequently, where ‘regularly’ meant exercising at least once a week. Smoking status was categorized as never smoked, ever smoked (quit) and currently smoked. Drinking status was classified as never (almost never drinkers), seldom (once a month to once every fortnight) and constant (once a week to once a day). Dorsal pedal artery pulsation was classified as bilateral palpable, non-palpable, and undetected. Ultrasound abdomen was classified as normal, abnormal, and undetected.

### 2.4 Statistical analysis

Continuous data were handled differently based on their distribution. For data following a normal distribution, values were expressed as mean ± standard deviation and analyzed using Analysis of Variance (ANOVA). For skewed data, values were expressed as median and quartiles and analyzed using the Kruskal-Wallis H test. Categorical data were expressed as frequencies and component ratios and analyzed using the chi-square test.

The normality of the lipid levels was assessed using the Kolmogorov–Smirnov test, which confirmed a skewed distribution. Accordingly, the Kruskal-Wallis H test was employed to evaluate variations in target indicators across groups that did not follow a normal distribution. Univariate analysis was utilized to identify statistically significant indicators, which, along with insights from other studies, informed the selection of variables for stepwise regression. This regression aimed to estimate the factors associated with the prevalence of dyslipidemia.

All statistical analyses were conducted using SPSS statistical software, Version 26.0 (IBM, New York, United States). Tests were two-sided and a *p*-value of less than 0.05 was considered statistically significant.

## 3 Results

### 3.1 The characteristics of participants

Table 1 describes the socio demographic and other characteristics of the hypertensives in this study. Of the 92,443 survey participants 48,661 (52.8%) were male and 43,582 (47.2%) were female, with mean ages of 61.30 ± 11.89, of which 59.23 ± 12.50 were males and 63.61 ± 10.70 were females, 96.0% were married, and 83.9% were non-domiciled. The prevalence of diabetes mellitus was 24.4%, coronary heart disease 5.4%, stroke 4.9%, and a lower prevalence of 0.8 and 0.3% for cancer and liver disease, respectively. The overall mean BMI was 25.13 ± 3.13, with overweight constituting 47.8% of the total respondents, normal weight 29.7%, obesity 21.3%, underweight 1.1%, and central obesity at nearly as high as 50%. In the general study population, the vast majority of patients were non-smokers (72.4%) and non-drinkers (78.6%), physically exercise everyday were 49.8%. The two main dorsalis pedis artery categories were bilateral symmetry (32.1%) and undetected (66.9%). The percentage of abnormal abdominal ultrasound was 35.5%. In terms of anthropometrics, the mean of systolic blood pressure, diastolic blood pressure, weight and waist circumference of the respondents were 140.84 ± 16.52 mmHg, 85.82 ± 11.20 mmHg, 65.34 ± 10.76 kg and 88.16 ± 9.37 cm, respectively. The total cholesterol, triglyceride, HDL cholesterol and LDL cholesterol levels were 4.94 ± 1.00 mmol/L, 1.58 ± 0.70 mmol/L, 1.28 ± 0.28 mmol/L and 2.97 ± 0.86 mmol/L correspondingly.

**Table 1:** Demographic and other characteristics of the study population.

Variables	All subjects (*N* = 92,443)	Male (*N* = 48,661)	Female (*N* = 43,582)	t/χ^2^	*P*
Age (years), mean ± SD	61.30 ± 11.886	59.23 ± 12.499	63.61 ± 10.699	57.425	<0.001
Age group (years), *n* (%)					
18–34	1,212 (1.3)	1,022 (2.1)	190 (0.4)	3615.054	<0.001
35–44	7,705 (8.4)	5,778 (11.9)	1927 (4.4)		
45–54	18,017 (19.5)	11,211 (23.0)	6,806 (15.6)		
55–64	22,150 (24.0)	10,807 (22.2)	11,343 (26.0)		
65–74	33,050 (35.8)	15,373 (31.6)	17,677 (40.6)		
75–84	8,534 (9.3)	3,800 (7.8)	4,734 (10.9)		
≥85	1,575 (1.7)	670 (1.4)	905 (2.1)		
Marital status, *n* (%)					
Married	88,599 (96.0)	46,707 (96.0)	41,892 (96.1)	1165.629	<0.001
Single	1,679 (1.8)	1,341 (2.8)	338 (0.8)		
Widowed	1,492 (1.6)	303 (0.6)	1,189 (2.7)		
Divorced	426 (0.5)	289 (0.6)	137 (0.3)		
Unspecified	47 (0.1)	21 (0.0)	26 (0.1)		
Resident status, *n* (%)					
Registered	14,851 (16.1)	8,133 (16.7)	6,718 (15.4)	28.719	<0.001
Non-registered	77,392 (83.9)	40,528 (83.3)	36,864 (84.6)		
Diabetes, *n* (%)					
Yes	22,479 (24.4)	11,562 (23.8)	10,917 (25.0)	20.727	<0.001
No	69,764 (75.6)	37,099 (76.2)	32,665 (75.0)		
Coronary heart disease, *n* (%)					
Yes	5,014 (5.4)	2,682 (5.5)	2,332 (5.4)	1.156	0.282
No	87,229 (94.6)	45,979 (94.5)	41,250 (94.6)		
Stroke, *n* (%)					
Yes	4,520 (4.9)	2,604 (5.4)	1916 (4.4)	44.996	<0.001
No	87,723 (95.1)	46,057 (94.6)	41,666 (95.6)		
Cancer, *n* (%)					
Yes	782 (0.8)	341 (0.7)	441 (1.0)	26.475	<0.001
No	91,461 (99.2)	48,320 (99.3)	43,141 (99.0)		
Liver disease, *n* (%)					
Yes	308 (0.3)	203 (0.4)	105 (0.2)	21.460	<0.001
No	91,935 (99.7)	48,458 (99.6)	43,477 (99.8)		
Family history of hypertension, *n* (%)					
Yes	31,231 (33.9)	16,496 (33.9)	14,735 (33.8)	0.083	0.773
No	61,012 (66.1)	32,165 (66.1)	28,847 (66.2)		
BMI categories, *n* (%)					
Underweight	1,060 (1.1)	473 (1.0)	587 (1.3)	601.650	<0.001
Normal	27,395 (29.7)	12,823 (26.4)	14,572 (33.4)		
Overweight	44,126 (47.8)	24,479 (50.3)	19,647 (45.1)		
Obese	19,662 (21.3)	10,886 (22.4)	8,776 (20.1)		
Central obesity, *n* (%)	44,702 (48.5)	23,041 (47.4)	21,661 (49.7)	50.908	<0.001
Physical exercise frequency, *n* (%)					
Daily	45,976 (49.8)	23,133 (47.5)	22,843 (52.4)	247.816	<0.001
Regularly	10,013 (10.9)	5,764 (11.8)	4,249 (9.7)		
Less frequently	36,254 (39.3)	19,764 (40.6)	16,490 (37.8)		
Smoking status, *n* (%)					
Non-smoker	66,759 (72.4)	23,630 (48.6)	43,129 (99.0)	29210.396	<0.001
Previous smoker	9,488 (10.3)	9,374 (19.3)	114 (0.3)		
Current smoker	15,996 (17.3)	15,657 (32.2)	339 (0.8)		
Alcohol consumption, *n* (%)					
Never	72,488 (78.6)	30,152 (62.0)	42,336 (97.1)	16930.608	<0.001
Seldom	11,585 (12.6)	10,679 (21.9)	906 (2.1)		
Constant	8,170 (8.9)	7,830 (16.1)	340 (0.8)		
Dorsal pedal artery pulsation, *n* (%)					
Bilateral palpable	29,655 (32.1)	15,048 (30.9)	14,607 (33.5)	71.718	<0.001
Non-palpable	911 (1.0)	475 (1.0)	436 (1.0)		
Undetected	61,677 (66.9)	33,138 (68.1)	28,539 (65.5)		
Abdominal ultrasound, *n* (%)					
Normal	21,249 (23.0)	10,435 (21.4)	10,814 (24.8)	761.880	<0.001
Abnormal	32,784 (35.5)	16,008 (32.9)	16,776 (38.5)		
Undetected	38,210 (41.4)	22,218 (45.7)	15,992 (36.7)		
BMI(kg/m^2^), mean ± SD	25.13 ± 3.13	25.29 ± 2.97	24.94 ± 3.29	−16.649	<0.001
Weight (kg), mean ± SD	65.34 ± 10.76	70.31 ± 9.81	59.78 ± 8.90	−171.002	<0.001
WC (cm), mean ± SD	88.16 ± 9.37	90.19 ± 8.06	85.88 ± 10.17	−70.753	<0.001
SBP (mmHg), mean ± SD	140.84 ± 16.52	140.45 ± 16.24	141.27 ± 16.83	7.511	<0.001
DBP (mmHg), mean ± SD	85.82 ± 11.20	87.70 ± 11.26	83.73 ± 10.75	−54.750	<0.001
TC (mmol/L), mean ± SD	4.94 ± 1.00	4.80 ± 0.98	5.09 ± 1.00	43.664	<0.001
TG (mmol/L), mean ± SD	1.58 ± 0.70	1.58 ± 0.72	1.57 ± 0.68	−3.901	<0.001
HDL-C (mmol/L), mean ± SD	1.28 ± 0.28	1.21 ± 0.27	1.35 ± 0.28	80.591	<0.001
LDL-C (mmol/L), mean ± SD	2.97 ± 0.86	2.93 ± 0.85	3.01 ± 0.87	15.450	<0.001

### 3.2 The prevalence of dyslipidemia component

Table 2 presents the dyslipidemia prevalence among different characteristics in gender, age, body size and other group. Dyslipidemia, high TC, high TG, low HDL-C and high LDL-C prevalence were 37.5, 11.2, 16.0, 16.0 and 10.2%, respectively. Among them, the prevalence of dyslipidemia, high TG, and low HDL-C was higher in men than in women, and the converse was for high TC and high LDL-C (all *p* < 0.001). The overweight, obesity, central obesity and infrequent exercise had a high prevalence of dyslipidemia and each of the subtypes of dyslipidemia. The prevalence of dyslipidemia increased progressively with the three smoking conditions of never smoker, former smoker (quit), and current smoker. A decrease in the prevalence of dyslipidemia was observed with age, and the prevalence of each subtype of dyslipidemia increased first and then decreased with age. The distribution characteristics of the numbers of dyslipidemia in gender and age are provided in Table 3. The number of subtypes with dyslipidemia was different between males and females and in different age. As shown in Table 4, lipid levels in abnormal grade was significantly different in gender. Men have far more low HDL-C than women and the proportion of exceeding the borderline high value of blood lipid was higher in women than that of men.

**Table 2:** Relationship between dyslipidemia prevalence and related factors.

Variables	Dyslipidemia^a^	High TC^1^	High TG^2^	Low HDL-C^3^	High LDL-C^4^
	*N* (%)	*N* (%)	*N* (%)	*N* (%)	*N* (%)
Gender, *n* (%)					
Male	19,670 (40.4)^***^	4,083 (8.4)^***^	8,170 (16.8)^***^	10,745 (22.1)^***^	4,403 (9.0)^***^
Female	14,896 (34.2)	6,261 (14.4)	6,547 (15.0)	3,985 (9.1)	4,993 (11.5)
Diabetes, *n* (%)					
Yes	9,173 (40.8)^***^	2,430 (10.8)^*^	4,049 (18.0)^***^	4,241 (18.9)^***^	2,257 (10.0)
No	25,393 (36.4)	7,914 (11.3)	10,668 (15.3)	10,489 (15.0)	7,139 (10.2)
Coronary heart disease, *n* (%)					
Yes	1782 (35.5)^***^	398 (7.9)^***^	685 (13.7)^***^	934 (18.6)^***^	358 (7.1)^***^
No	32,784 (37.6)	9,946 (11.4)	14,032 (16.1)	13,796 (15.8)	9,038 (10.4)
Stroke, *n* (%)					
Yes	1,561 (34.5)^***^	356 (7.9)^***^	575 (12.7)^***^	827 (18.3)^***^	335 (7.4)^***^
No	33,005 (37.6)	9,988 (11.4)	14,142 (16.1)	13,903 (15.8)	9,061 (10.3)
Cancer, *n* (%)					
Yes	281 (35.9)	80 (10.2)	110 (14.1)	115 (14.7)	77 (9.8)
No	34,285 (37.5)	10,264 (11.2)	14,607 (16.0)	14,615 (16.0)	9,319 (10.2)
Liver disease, *n* (%)					
Yes	118 (38.3)	30 (9.7)	39 (12.7)	63 (20.5)^*^	30 (9.7)
No	34,448 (37.5)	10,314 (11.2)	14,678 (16.0)	14,667 (16.0)	9,366 (10.2)
Age group (years), *n* (%)					
18–34	523 (43.2)^***^	105 (8.7)^***^	280 (23.1)^***^	251 (20.7)^***^	116 (9.6)^***^
35–44	3,375 (43.8)	674 (8.7)	1777 (23.1)	1,669 (21.7)	739 (9.6)
45–54	7,149 (39.7)	1,601 (8.9)	3,376 (18.7)	3,384 (18.8)	1,664 (9.2)
55–64	8,172 (36.9)	2,591 (11.7)	3,482 (15.7)	3,270 (14.8)	2,339 (10.6)
65–74	11,871 (35.9)	4,135 (12.5)	4,586 (13.9)	4,677 (14.2)	3,538 (10.7)
75–84	2,975 (34.9)	1,074 (12.6)	1,064 (12.5)	1,217 (14.3)	877 (10.3)
≥85	501 (31.8)	164 (10.4)	152 (9.7)	262 (16.6)	123 (7.8)
Family history of hypertension, *n* (%)					
Yes	12,013 (38.5)^***^	3,364 (10.8)^**^	5,286 (16.9)^***^	5,154 (16.5)^**^	3,001 (9.6)^***^
No	22,553 (37.0)	6,980 (11.4)	9,431 (15.5)	9,576 (15.7)	6,395 (10.5)
BMI categories, *n* (%)					
Underweight	197 (18.6)^***^	104 (9.8)	43 (4.1)^***^	60 (5.7)^***^	74 (7.0)^***^
Normal	8,169 (29.8)	3,048 (11.1)	2,847 (10.4)	3,025 (11.0)	2,560 (9.3)
Overweight	17,383 (39.4)	5,041 (11.4)	7,489 (17.0)	7,580 (17.2)	4,625 (10.5)
Obese	8,817 (44.8)	2,151 (10.9)	4,338 (22.1)	4,065 (20.7)	2,137 (10.9)
Central obesity, *n* (%)					
Normal	15,499 (32.6)^***^	5,095 (10.7)^***^	5,909 (12.4)^***^	6,318 (13.3)^***^	4,448 (9.4)^***^
Abdominal obesity	19,067 (42.7)	5,249 (11.7)	8,808 (19.7)	8,412 (18.8)	4,948 (11.1)
Physical exercise frequency, *n* (%)					
Daily	16,509 (35.9)^***^	5,332 (11.6)^***^	6,438 (14.0)^***^	6,922 (15.1)^***^	4,726 (10.3)
Regularly	3,861 (38.6)	1,110 (11.1)	1722 (17.2)	1722 (17.2)	987 (9.9)
Less frequently	14,196 (39.2)	3,902 (10.8)	6,557 (18.1)	6,086 (16.8)	3,683 (10.2)
Smoking status, *n* (%)					
Non-smoker	23,720 (35.5)^***^	8,159 (12.2)^***^	10,156 (15.2)^***^	8,771 (13.1)^***^	7,031 (10.5)^***^
Previous smoker	3,669 (38.7)	752 (7.9)	1,351 (14.2)	2034 (21.4)	777 (8.2)
Current smoker	7,177 (44.9)	1,433 (9.0)	3,210 (20.1)	3,925 (24.5)	1,588 (9.9)
Alcohol consumption, *n* (%)					
Never	26,694 (36.8)^***^	8,487 (11.7)^***^	11,165 (15.4)^***^	10,894 (15.0)^***^	7,536 (10.4)^***^
Seldom	4,772 (41.2)	1,085 (9.4)	2,119 (18.3)	2,459 (21.2)	1,131 (9.8)
Constant	3,100 (37.9)	772 (9.4)	1,433 (17.5)	1,377 (16.9)	729 (8.9)

*:*p* < 0.05; **:*p* < 0.01; ***:*p* < 0.001. 1. TC: Total cholesterol; 2. TG: Triglyceride; 3. HDL-C: High-density lipoprotein cholesterol; 4. LDL-C: Low-density lipoprotein cholesterol. **The existence of either High TC, High TG, Low HDL-C, High LDL-C is considered dyslipidaemia**.

**Table 3:** Distribution charactristics of several dyslipidemia.

	Number of subtypes of dyslipidemia					*P* value
	0	1	2	3	4	
Gender						
Male	28,991 (59.6)_a_	13,070 (26.9)_b_	5,593 (11.5)_c_	883 (1.8)_a,c_	124 (0.3)_d_	<0.001
Female	28,686 (65.8)_a_	9,002 (20.7)_b_	4,951 (11.4)_c_	890 (2.0)_a,c_	53 (0.1)_d_	
Age group (years)						
18–34	689 (56.8)_a_	332 (27.4)_b_	157 (13.0)_a,b_	30 (2.5)_a,b_	4 (0.3)_a,b_	<0.001
35–44	4,330 (56.2)_a_	2,135 (27.7)_b_	1,029 (13.4)_b_	178 (2.3)_b_	33 (0.4)_c_	
45–54	10,868 (60.3)_a_	4,714 (26.2)_b_	2033 (11.3)_a_	363 (2.0)_a,b_	39 (0.2)_a,b_	
55–64	13,978 (63.1)_a_	5,169 (23.3)_a_	2,535 (11.4)_a_	429 (1.9)_a_	39 (0.2)_a_	
65–74	21,179 (64.1)_a_	7,507 (22.7)_b_	3,704 (11.2)_b_	619 (1.9)_a,b_	41 (0.1)_c_	
75–84	5,559 (65.1)_a_	1884 (22.1)_b_	940 (11.0)_a,b_	136 (1.6)_a,b_	15 (0.2)_a,b_	
≥85	1,074 (68.2)_a_	331 (21.0)_b_	146 (9.3)_b_	18 (1.1)_a,b_	6 (0.4)_a,b_	

**Table 4:** Proportion of lipid fraction serum levels.

	Overall	Gender		
		Male	Female	*P* value
Total cholesterol (mmol/L)				
Desirable (<5.18)	55,446 (60.1)	31,852 (65.5)	23,594 (54.1)	<0.001
Borderline high (5.18- < 6.22)	26,858 (29.1)	12,907 (26.5)	13,951 (32.0)	
High (≥6.22)	9,939 (10.8)	3,902 (8.0)	6,037 (13.9)	
Triglycerides (mmol/L)				
Desirable (<1.69)	58,600 (63.5)	30,640 (63.0)	27,960 (64.2)	<0.001
Borderline high (1.69–2.26)	18,107 (19.6)	9,398 (19.3)	8,709 (20.0)	
High (≥2.26)	15,536 (16.8)	8,623 (17.7)	6,913 (15.9)	
LDL-C (mmol/L)				
Desirable (<2.59)	30,943 (33.5)	17,043 (35.0)	13,900 (31.9)	<0.001
Near or above optimal (2.59- < 3.37)	31,835 (34.5)	17,006 (34.9)	14,829 (34.0)	
Borderline high (3.37- < 4.14)	20,814 (22.6)	10,594 (21.8)	10,220 (23.5)	
High (4.14- < 4.92)	7,441 (8.1)	3,470 (7.1)	3,971 (9.1)	
Very high (≥4.92)	1,210 (1.3)	548 (1.1)	662 (1.5)	
HDL-C (mmol/L)				
Low (<1.04)	19,153 (20.8)	13,683 (28.1)	5,470 (12.6)	<0.001
Desirable (1.04- < 1.55)	56,935 (61.7)	29,394 (60.4)	27,541 (63.2)	
High (≥1.55)	16,155 (17.5)	5,584 (11.5)	10,571 (24.3)	

### 3.3 The lipid level of dyslipidemia component

Table 5 shows the subtype of dyslipidemia levels among various characteristic group. The levels of high total cholesterol, high triglyceride, low HDL cholesterol, and high LDL cholesterol abnormality differed significantly between genders in respondents (*p* < 0.001), and the levels of them were all higher in females. Hypertensive patients with diabetic had higher TG levels than non-diabetic (*p* = 0.009), with stroke had lower levels than non-stroke (*p* = 0.018), and with liver disease had lower levels than non-liver disease patients (*p* < 0.001). In the dyslipidemia population with low HDL-C levels, HDL-C levels were lower in diabetic patients than in non-diabetic patients (*p* < 0.001), in patients with coronary artery disease than in non-coronary artery disease (*p* = 0.026), and in centrally obesity than in non-centrally obesity (*p* < 0.001). The variation in HDL-C levels among the four categories of BMI was statistically significant (*p* = 0.005), and by further two-by-two comparisons between groups, overweight and obese had lower HDL-C levels than normal weight. LDL-C levels were lower in non-centrally obese than in centrally obese (*p* = 0.018). TC levels increased and then decreased with age (*p* < 0.001), highest in the 75–84 years age group. In the high TG abnormalities, significant difference was observed among different drinking frequency (*p* = 0.004), and its levels increased with the frequency of alcohol consumption. TG levels declined gradually with aging (*p* < 0.001). By further two-by-two comparisons between groups, overweight and obese had higher TG levels than normal weight (*p* = 0.015), less frequently exercised had higher TG levels than daily exercised (*p* = 0.015).

**Table 5:** Relationship between dyslipidemia constituents levels and related factors.

Variables	*N*	High TC^1^			High TG^2^			Low HDL-C^3^			High LDL-C^4^		
		*n*	Median	IQR^5^	*n*	Median	IQR	*n*	Median	IQR	*n*	Median	IQR
Gender													
Male	48,661	4,083	6.56^***^	6.35–6.88	8,170	2.71^***^	2.48–3.09	10,745	0.90^***^	0.84–0.95	4,403	4.42^*^	4.23–4.70
Female	43,582	6,261	6.62	6.38–6.96	6,547	2.75	2.49–3.13	3,985	0.92	0.86–0.96	4,993	4.44	4.24–4.71
Diabetes													
Yes	22,479	2,430	6.61	6.38–6.94	4,049	2.75^**^	2.50–3.13	4,241	0.90^***^	0.83–0.95	2,257	4.43	4.24–4.72
No	69,764	7,914	6.59	6.37–6.92	10,668	2.73	2.48–3.10	10,489	0.91	0.85–0.96	7,139	4.43	4.24–4.70
Coronary heart disease													
Yes	5,014	398	6.59	6.39–6.93	685	2.69	2.47–3.09	934	0.90^*^	0.84–0.95	358	4.44	4.24–4.70
No	87,229	9,946	6.60	6.37–6.92	14,032	2.74	2.49–3.11	13,796	0.91	0.85–0.96	9,038	4.43	4.24–4.70
Stroke													
Yes	4,520	356	6.61	6.40–6.97	575	2.71^*^	2.47–3.04	827	0.91	0.84–0.96	335	4.43	4.25–4.70
No	87,723	9,988	6.60	6.37–6.92	14,142	2.74	2.49–3.11	13,903	0.91	0.85–0.96	9,061	4.43	4.24–4.70
Cancer													
Yes	782	80	6.65^*^	6.43–7.14	110	2.69	2.48–3.05	115	0.90	0.84–0.96	77	4.50	4.28–4.80
No	91,461	10,264	6.60	6.37–6.92	14,607	2.74	2.49–3.11	14,615	0.91	0.84–0.96	9,319	4.43	4.24–4.70
Liver disease													
Yes	308	30	6.58	6.38–6.76	39	2.49^***^	2.35–2.84	63	0.90	0.84–0.95	30	4.39	4.25–4.57
No	91,935	10,314	6.60	6.37–6.92	14,678	2.74	2.49–3.11	14,667	0.91	0.84–0.96	9,366	4.43	4.24–4.70
Age group (years)													
18–34	1,212	105	6.51^***^	6.34–6.82	280	2.81^***^	2.50–3.18	251	0.91	0.85–0.95	116	4.43	4.20–4.77
35–44	7,705	674	6.52	6.32–6.84	1777	2.81	2.51–3.20	1,669	0.91	0.85–0.96	739	4.42	4.24–4.70
45–54	18,017	1,601	6.56	6.35–6.90	3,376	2.75	2.49–3.14	3,384	0.91	0.84–0.96	1,664	4.40	4.22–4.69
55–64	22,150	2,591	6.60	6.38–6.95	3,482	2.73	2.50–3.12	3,270	0.91	0.85–0.96	2,339	4.43	4.24–4.70
65–74	33,050	4,135	6.61	6.38–6.94	4,586	2.70	2.48–3.08	4,677	0.91	0.84–0.96	3,538	4.43	4.24–4.72
75–84	8,534	1,074	6.62	6.38–6.95	1,064	2.67	2.47–3.01	1,217	0.90	0.83–0.96	877	4.45	4.25–4.72
≥85	1,575	164	6.58	6.38–6.89	152	2.63	2.47–2.97	262	0.90	0.83–0.95	123	4.40	4.24–4.67
Family history of hypertension													
Yes	31,231	3,364	6.58	6.37–6.91	5,286	2.74	2.49–3.13	5,154	0.91	0.84–0.96	3,001	4.43	4.24–4.69
No	61,012	6,980	6.61	6.37–6.93	9,431	2.73	2.49–3.10	9,576	0.91	0.85–0.96	6,395	4.43	4.24–4.71
BMI categories													
Underweight (<18.5)	1,060	104	6.62	6.39–6.97	43	2.64^*^	2.54–3.03	60	0.90^**^	0.82–0.96	74	4.41	4.26–4.68
Normal (18.5–24.0)	27,395	3,048	6.61	6.37–6.92	2,847	2.71	2.48–3.08	3,025	0.91	0.85–0.96	2,560	4.43	4.23–4.70
Overweight (24.0–28.0)	44,126	5,041	6.60	6.38–6.93	7,489	2.74	2.49–3.11	7,580	0.91	0.85–0.95	4,625	4.43	4.24–4.70
Obese (≥28.0)	19,662	2,151	6.57	6.36–6.92	4,338	2.74	2.49–3.13	4,065	0.91	0.84–0.96	2,137	4.42	4.23–4.71
Central obesity													
Normal	47,541	5,095	6.59	6.37–6.92	5,909	2.73	2.49–3.10	6,318	0.91^***^	0.85–0.96	4,448	4.42^*^	4.23–4.69
Abdominal obesity	44,702	5,249	6.60	6.38–6.93	8,808	2.74	2.49–3.12	8,412	0.91	0.84–0.95	4,948	4.43	4.24–4.72
Physical exercise frequency													
Daily	45,976	5,332	6.60	6.38–6.92	6,438	2.72^**^	2.49–3.09	6,922	0.91	0.84–0.96	4,726	4.43	4.24–4.71
Regularly	10,013	1,110	6.59	6.38–6.92	1722	2.73	2.47–3.11	1722	0.91	0.85–0.96	987	4.43	4.23–4.69
Less frequently	36,254	3,902	6.60	6.36–6.93	6,557	2.76	2.49–3.13	6,086	0.91	0.84–0.96	3,683	4.42	4.24–4.71
Smoking status													
Non-smoker	66,759	8,159	6.61^***^	6.38–6.94	10,156	2.73^***^	2.48–3.10	8,771	0.91^***^	0.85–0.96	7,031	4.43	4.24–4.70
Previous smoker	9,488	752	6.55	6.36–6.83	1,351	2.72	2.48–3.09	2034	0.91	0.84–0.95	777	4.43	4.23–4.71
Current smoker	15,996	1,433	6.56	6.36–6.88	3,210	2.77	2.50–3.16	3,925	0.90	0.83–0.95	1,588	4.42	4.23–4.70
Alcohol consumption													
Never	72,488	8,487	6.60	6.37–6.93	11,165	2.73^**^	2.49–3.10	10,894	0.91	0.85–0.96	7,536	4.43	4.24–4.71
Seldom	11,585	1,085	6.59	6.37–6.91	2,119	2.76	2.50–3.13	2,459	0.91	0.85–0.96	1,131	4.40	4.24–4.68
Constant	8,170	772	6.58	6.35–6.91	1,433	2.78	2.50–3.15	1,377	0.91	0.84–0.95	729	4.44	4.24–4.71

*:*P* < 0.05; **:*P* < 0.01; ***:*P* < 0.001. 1. TC: Total cholesterol; 2. TG: Triglyceride; 3. HDL-C: High-density lipoprotein cholesterol; 4. LDL-C: Low-density lipoprotein cholesterol; 5. IQR stands for Interquartile Range.

### 3.4 The prevalence and influencing factors of dyslipidemia

The prevalence of dyslipidemia in this study respondents was 37.5%. A univariate logistic regression model was used to evaluate multiple potentially independent factors that may influence the prevalence of dyslipidemia. The results indicate that the variables gender, age, marital status, diabetes, coronary heart disease, stroke, family history of hypertension, BMI category, central obesity, dorsal pedal artery pulsation, abdominal ultrasound, smoking status, alcohol consumption, and physical exercise frequency were significantly associated with the prevalence of dyslipidemia (Table 6). However, statistically significant differences in the prevalence of dyslipidemia were not found between resident status, cancer and liver disease. Subsequently, multiple stepwise regression analysis confirmed these associations, with the addition of undetected dorsal pedal artery pulsation and constant alcohol consumption as significant factors. Findings showed a gradual decrease in the risk of dyslipidemia with increasing age. Among the four categories of BMI, the risk of dyslipidemia increased with increasing body size (underweight: OR = 0.571, *p* < 0.001; overweight: OR = 1.318, *p* < 0.001; obesity: OR = 1.444, *p* < 0.001). Compared to daily physical exercise frequency, the risk of dyslipidemia was higher as the frequency of exercise decreased (regularly: OR = 1.059, *p* = 0.014; less frequently: OR = 1.078, *p* < 0.001). The prevalence of dyslipidemia as compared to non-smokers was elevated by 1.091-fold (*p* = 0.001) and 1.354-fold (*p* < 0.001) in ex-smokers and current smokers, respectively. The dyslipidemia prevalence was higher in those with abnormal abdominal ultrasound and those who were undetected compared to those with normal abdominal ultrasound results (abnormal: OR = 1.409, *p* < 0.001; undetected: OR = 1.395, *p* < 0.001). Prevalence of dyslipidemia are 1.187 multiples higher in diabetic patients (*p* < 0.001), and the risk of dyslipidemia in patients with comorbid coronary heart disease and stroke declined (coronary heart disease: OR = 0.902, *p* < 0.001; stroke: OR = 0.878, *p* < 0.001).

**Table 6:** Multivariate stepwise logistic regression analysis of factors associated with dyslipidemia.

Variables	OR (95% CI)	*P* value	m_OR (95% CI)	m_*P* value
Gender	1.307 (1.272–1.342)	0.000	1.165 (1.125–1.206)	0.000
Age group (years)				
18–34	1 (reference)		1 (reference)	
35–44	1.027 (0.909–1.160)	0.671	1.015 (0.896–1.149)	0.817
45–54	0.867 (0.771–0.975)	0.017	0.883 (0.784–0.996)	0.042
55–64	0.770 (0.685–0.866)	0.000	0.828 (0.735–0.933)	0.002
65–74	0.738 (0.658–0.829)	0.000	0.832 (0.737–0.940)	0.003
75–84	0.705 (0.624–0.797)	0.000	0.812 (0.714–0.923)	0.001
≥85	0.615 (0.526–0.718)	0.000	0.748 (0.637–0.879)	0.000
Marital status				
Married	1 (reference)			
Single	1.172 (1.063–1.293)	0.001		
Widowed	0.871 (0.782–0.970)	0.012		
Divorced	1.308 (1.080–1.585)	0.006		
Unspecified	1.239 (0.695–2.209)	0.468		
Resident status	0.976 (0.941–1.012)	0.183		
Diabetes	1.205 (1.168–1.242)	0.000	1.187 (1.144–1.231)	0.000
Coronary heart disease	0.916 (0.863–0.972)	0.004	0.902 (0.847–0.959)	0.001
Stroke	0.875 (0.821–0.931)	0.000	0.878 (0.823–0.937)	0.000
Cancer	0.935 (0.808–1.083)	0.372		
Liver disease	1.036 (0.823–1.305)	0.761		
Family history of hypertension	1.066 (1.036–1.096)	0.000	1.039 (1.009–1.070)	0.010
BMI categories				
Underweight (<18.5)	0.537 (0.459–0.629)	0.000	0.571 (0.487–0.668)	0.000
Normal (18.5–24.0)	1 (reference)		1 (reference)	
Overweight (24.0–28.0)	1.530 (1.481–1.580)	0.000	1.318 (1.272–1.366)	0.000
Obese (≥28.0)	1.913 (1.842–1.988)	0.000	1.444 (1.376–1.516)	0.000
Central obesity	1.538 (1.497–1.579)	0.000	1.250 (1.209–1.293)	0.000
Dorsal pedal artery pulsation				
Bilateral palpable	1 (reference)		1 (reference)	
Non-palpable	1.077 (0.942–1.232)	0.276	1.077 (0.940–1.235)	0.284
Undetected	0.910 (0.884–0.936)	0.000	0.936 (0.905–0.969)	0.000
Abdominal ultrasound				
Normal	1 (reference)		1 (reference)	
Abnormal	1.524 (1.469–1.581)	0.000	1.345 (1.295–1.397)	0.000
Undetected	1.449 (1.398–1.501)	0.000	1.203 (1.153–1.255)	0.000
Smoking status				
Non-smoker	1 (reference)		1 (reference)	
Previous smoker	1.144 (1.095–1.196)	0.000	1.091 (1.037–1.148)	0.001
Current smoker	1.477 (1.426–1.529)	0.000	1.354 (1.298–1.413)	0.000
Alcohol consumption				
Never	1 (reference)		1 (reference)	
Seldom	1.202 (1.155–1.251)	0.000	0.961 (0.920–1.005)	0.080
Constant	1.049 (1.001–1.100)	0.047	0.846 (0.803–0.891)	0.000
Physical exercise frequency				
Daily	1 (reference)		1 (reference)	
Regularly	1.120 (1.071–1.171)	0.000	1.059 (1.011–1.109)	0.014
Less frequently	1.149 (1.117–1.182)	0.000	1.078 (1.045–1.111)	0.000

## 4 Discussion

The analysis of this study is notable for the comparison of lipid levels and the prevalence of the corresponding dyslipidemia components in different categorical characteristics groups. Not only does it allow comparison of the likelihood of dyslipidemia in different groups, but it also analyses the degree of dyslipidemia in the context of lipid levels to better analyze how much higher the dyslipidemia level are in different categories. Comparisons of the overall prevalence of dyslipidemia presenting any lipid component abnormality were made in the various indicators. Differences in blood lipids were analysed between socio-demographic, behavioral and lifestyle characteristics such as gender, age, co-morbidities, family history of hypertension, BMI classification, central obesity, frequency of physical activity, smoking status, alcohol use, abdominal ultrasound and dorsalis pedis arterial results, and physiological and biochemical findings, and stepwise regression was used to investigate the factors affecting the prevalence of blood lipids.

The population investigated in this study was mainly middle-aged and older adult. The prevalence of dyslipidemia in this study are higher than the results of the prevalence of dyslipidemia among adults in northern China (31.2%) reported by Xi et al. (11), lower than the overall prevalence of dyslipidemia among older adults in outheast coastal regions in China (27.21%) investigated by Lin et al. (12), and higher than the results of the rate of dyslipidemia among older adult people in Anning City, Yunnan Province (33.81%) by Lu et al. (13), slightly lower than the results of Chaohong et al. on the rate of hypertension combined with dyslipidemia among hypertensive residents of Zhengzhou City (37.8%) (14). It was found that the prevalence of dyslipidemia in hypertensive patients in Shenzhen was lower than the Chinese prevalence of dyslipidemia in adults (40.4%) (15), and also lower than the prevalence of dyslipidemia in hyperpietic of other regions. The main types of dyslipidemia were high TC abnormality and low HDL-C abnormality, consistent with the results of other studies (16). Concordant with the results of the 2018 study on the prevalence of dyslipidemia in adult residents of Hebei Province conducted by Yajing et al (17), the prevalence of high TG (15.28%) and low HDL (22.30%) was higher than the prevalence of high TC (6.62%) and high LDL (5.01%). However, unlike the results of the dyslipidemia analysis of older adult residents in Shanghai by Weilan et al. (18), the prevalence of high TC (17.0%) and high LDL-C (12.5%) in this study was higher than high TC (6.4%) and low HDL-C (5.1%), which included elders and predominantly farmer population with higher physical activity and more sensible dietary habits, leading to lipid profile results different from other studies.

NCD-RisC (19) study showed that developing countries, especially China, is one of the fastest rates of increase in non-HDL-C levels in the world over the last 40 years (20). This study indicated that high TG was the main type of dyslipidemia in males (16.8%) and high TC was the main type of dyslipidemia in females (14.4%); however, among the high TG population, the TG level in females was higher than that in males. Consistent with the type of abnormality in Jian et al.’s (21) study on the current status of lipids in the older adult in Guangdong Province, and with the results of the study by Huang Ei-Hsian et al. about dyslipidemia in older adult community residents in Guangzhou. The prevalence of dyslipidemia in the study population decreased with increasing age, but the levels of each dyslipidemia increased and then decreased with age. In the present study, the levels of high TC, high TG, low HDL-C, and high LDL-C were higher in the age group 75–84 than in the age group 85 and above, which is in accordance with the results of several studies (22). It may be related to the increasing functional deterioration in the older adult, especially in the digestive tract and lipid metabolism functions, which leads to a decrease in dyslipidemia levels with age (23). The respondents included in this study were unselected and included a proportion of patients with dyslipidemia and cardiovascular disease, some of whom were taking lipid-modifying drugs, which can have an impact on their lipid levels.

Exercise can reduce the prevalence of high TG and the concentration of abnormal TG levels. As the frequency of exercise decreases, the prevalence of dyslipidemia increases in Shenzhen hypertensive patients. Regular physical exercise of appropriate intensity can effectively promote the body’s metabolism, improve the activity of lipoprotein esterase, thus accelerating the decomposition and excretion of fat; regular exercise can also play a role in improving the body’s glucose metabolism, which is conducive to improving the state of blood coagulation, then blood viscosity can reduced to a certain extent; reasonable and regular physical exercise can enhance the metabolism of cardiomyopathy, improve the function of cardiac muscle, and has positive significance in the prevention and treatment of dyslipidemia and cardiovascular diseases. Smoking increases the prevalence of low HDL-C and the concentration of abnormal HDL-C levels decreased. Smoking leads to hardening of the blood vessels in the brain, promotes blood clotting and exacerbates vascular lesions, thereby increasing the probability of dyslipidemia and stroke (24). The prevalence of dyslipidemia was higher in frequent drinkers than in never drinkers, suggesting that alcohol consumption is a risk factor for dyslipidemia. However, the relationship between alcohol consumption and dyslipidemia is complex and may depend on various factors such as the amount and type of alcohol consumed, genetic factors, and overall lifestyle. Mixue et al. (25) and Li et al. (9) showed that alcohol consumption may be a protective factor for dyslipidemia. Our findings seem to contradict this, which highlights the need for more detailed research on alcohol consumption patterns and their effects on lipid profiles. The frequency of alcohol consumption was not investigated in this study, so it cannot be excluded that frequent drinkers pay more attention to other health behaviors, such as healthier dietary habits and controlling the amount of alcohol consumed per drinking occasion, whereas the prevalence of dyslipidemia was higher in the less frequent drinkers than in the regular drinkers, although the frequency of alcohol consumption was less, but the amount of alcohol consumed per occasion may be high and due to the lack of health awareness and inappropriate behavioral lifestyles. Klop et al. study suggests that mild to moderate alcohol consumption may be associated with lower lipid, which may depend on the type of alcoholic beverage consumed, genetic polymorphisms, and lifestyle factors (26). Mansour et al. (27) and Lan-Ling (28) pointed out that the relationship between alcohol consumption and dyslipidemia is still not clear, but the long-term and large amount of alcohol consumption may easily lead to various kinds of alcoholic liver diseases, which further cause TG and TC metabolic disorders, increasing the risk of cardiovascular disease, alcohol drinking is not recommended as a preventive or therapeutic measure for dyslipidemia (29–31).

In this study, TG levels were higher in patients with combined diabetes and combined liver disease than in patients with non-diabetes and non-liver disease; TG levels were higher in non-stroke patients than in stroke patients, suggesting that lipid levels in patients with cardiovascular disease had been controlled and improved; high TG levels were negatively correlated with age, and positively correlated with BMI category, physical activity frequency, and alcohol consumption. High LDL-C levels were associated with gender and central obesity. Low HDL-C levels were associated with diabetes, coronary heart disease, BMI, central obesity, and smoking status. Diabetes is the risk factor for comorbid dyslipidemia in hypertensive patients in Shenzhen, consistent with previous studies (32, 33). It is speculated that the presence of insulin resistance in diabetic patients may contribute to the development of hypertension and hyperlipidemia (34), which suggests that control and prevention of diabetes and other related chronic diseases when preventing and controlling hypertension combined with dyslipidemia (35, 36). Currently, dyslipidemia is prevalent but management rate is low, and a huge gap still exists in the health management of dyslipidemia. Targeted lipid management measures should be taken to improve the effectiveness and comprehensiveness of lipid management in hypertensive patients in the community.

The limitations of this study should be mentioned. The disease history and behavioral information were self-reported by the respondents, and recall bias may have existed. Diet and medication use were not included in the comparative analysis, which may have influenced the lipids and study results. The absence of dorsalis pedis arterial pulsation and abdominal ultrasound constituted the majority of patients, which could not fully support the association between abnormal dorsalis pedis arterial pulsation and abdominal ultrasound and the incidence of dyslipidemia. Lastly, this study is a cross-sectional design, which can only propose relevant etiological hypotheses and cannot directly infer causality.

## 5 Conclusion

The prevalence of dyslipidemia among the respondents was 37.5% in this study, high TC was the most frequent form of dyslipidemia among the participants, followed by low level of HDL. The lipid levels of hypertensive patients should receive more attention, understanding the lipid profiles of hypertensive patients can help in clinical treatment and the development of more aggressive public health measures and screening programmes to promote primary prevention interventions, avoiding further progression of patients to cardiovascular and cerebral vascular diseases such as stroke, atherosclerotic heart disease, cerebral hemorrhage, etc. On the implementation of basic public health services for hypertension and diabetes mellitus, promote the development and implementation of health management services for dyslipidemia. Future studies should consider exploring the relationship between dyslipidemia and kidney disease in hypertensive patients, as well as investigating the complex relationship between alcohol consumption and lipid profiles in more detail.

## Data Availability

The original contributions presented in the study are included in the article/supplementary material, further inquiries can be directed to the corresponding author.
